# Supplementary figures and images for: Structure–function analysis of Lactiplantibacillus plantarum DltE reveals D-alanylated lipoteichoic acids as direct cues supporting Drosophila juvenile growth
Source: eLife. 2023 Apr 12;12:e84669. doi: 10.7554/eLife.84669 (PMC10241514; doi:10.7554/eLife.84669)

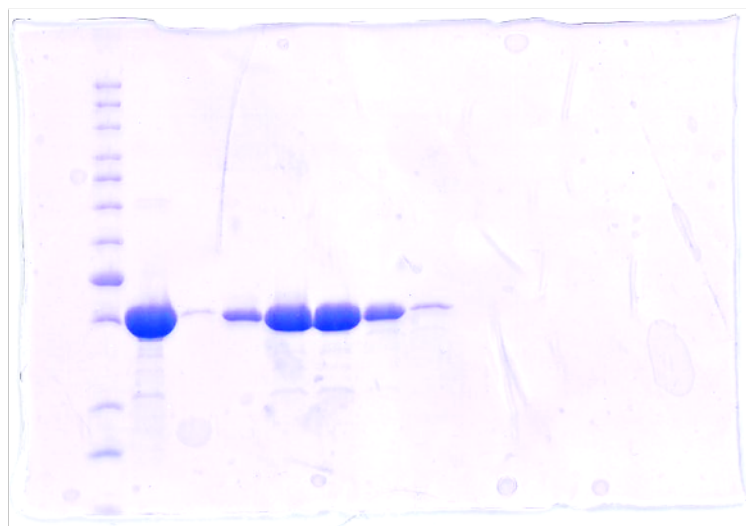

Supplement: Figure 1—source data 2. [file elife-84669-fig1-data2.pdf]

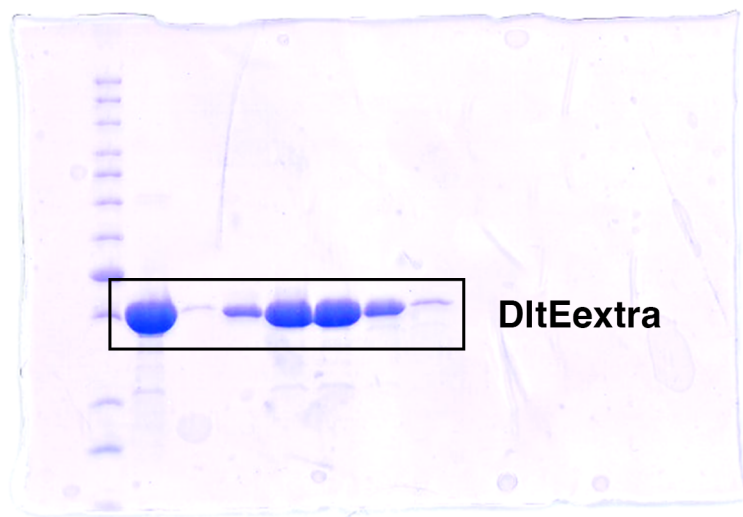

Supplement: Figure 1—source data 3. [file elife-84669-fig1-data3.pdf]

NC8     $\Delta dlt_{op}$      $\Delta tagO$      $\Delta taS$

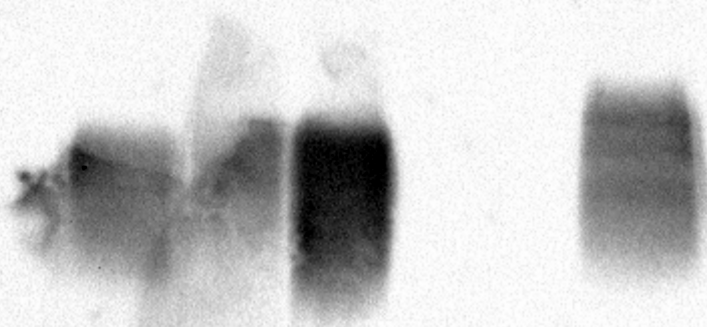

Supplement: Figure 5—source data 4. [file elife-84669-fig5-data4.pdf]
