# Supplementary material for: Structure–function analysis of Lactiplantibacillus plantarum DltE reveals D-alanylated lipoteichoic acids as direct cues supporting Drosophila juvenile growth
Source: eLife. 2023 Apr 12;12:e84669. doi: 10.7554/eLife.84669 (PMC10241514; doi:10.7554/eLife.84669)
Supplement: Supplementary file 1. [file elife-84669-supp1.docx]

**Supplementary Table 1.** Data collection and refinement statistics.

|  | DltE _extra_-apo | DltE _extra_-tartrate | DltE _extra_-TCEP | DltE _extra_-TA |
| --- | --- | --- | --- | --- |
| **Crystallization conditions** | 26% PEG MME 5K, 0.1M Tris pH8.5, 0.15M LiSO_4_ and 30% ethylene glycol | 26% PEG 3350, 0.1M ammonium tartrate | 20% PEG 3350, 0.2M ammonium tartrate and 0.1M TCEP HCl | 26% PEG 3350, 0.1M ammonium tartrate  24h soaking with 10mM LTA |
| **Wavelength** | 0.87 | 0.97 | 0.87 | 0.98 |
| **Resolution range** | 47.75 - 1.86 (1.926 - 1.86) | 41.04 - 1.95 (2.02 - 1.95) | 49.45 - 1.94 (2.009 - 1.94) | 46.33 - 1.4  (1.45 - 1.4) |
| **Space group** | P 1 21 1 | P 21 21 21 | P 1 21 1 | P 21 21 21 |
| **Number of molecules in the a.u.** | 2^1^ | 2^2^ | 4^3^ | 2^4^ |
| **Unit cell (Å, °)** | 60.87, 95.712, 68.01, 90, 96.26, 90 | 48.69, 103.73, 152.53, 90, 90, 90 | 76.24, 98.64, 135.93, 90, 105.79, 90 | 48.68, 96.07, 150.83, 90, 90, 90 |
| **Total reflections** | 128187 (12709) | 113125 (11117) | 268566 (25402) | 279608 (27536) |
| **Unique reflections** | 64925 (6451) | 56870 (5568) | 139733 (13635) | 139817 (13770) |
| **Multiplicity** | 2.0 (2.0) | 2.0 (2.0) | 1.9 (1.9) | 2.0 (2.0) |
| **Completeness (%)** | 99.81 (99.92) | 99.32 (99.70) | 97.57 (95.26) | 99.98 (99.96) |
| **Mean I/sigma(I)** | 6.50 (2.16) | 15.08 (2.60) | 5.36 (2.49) | 16.61 (1.68) |
| **Wilson B-factor** | 14.15 | 32.35 | 21.70 | 15.31 |
| **R-merge** | 0.08491 (0.296) | 0.02214 (0.2728) | 0.06333 (0.2724) | 0.02281 (0.4422) |
| **R-meas** | 0.1201 (0.4186) | 0.03132 (0.3858) | 0.08957 (0.3853) | 0.03226 (0.6253) |
| **R-pim** | 0.08491 (0.296) | 0.02214 (0.2728) | 0.06333 (0.2724) | 0.02281 (0.4422) |
| **CC1/2** | 0.988 (0.791) | 0.999 (0.852) | 0.99 (0.822) | 1 (0.678) |
| **CC*** | 0.997 (0.94) | 1 (0.959) | 0.997 (0.95) | 1 (0.899) |
| **Reflections used in refinement** | 64911 (6451) | 56861 (5567) | 139689 (13635) | 139809 (13766) |
| **Reflections used for R-free** | 3142 (288) | 2810 (293) | 1993 (186) | 1053 (103) |
| **R-work** | 0.1715 (0.2609) | 0.1877 (0.3129) | 0.1869 (0.2420) | 0.1742 (0.2679) |
| **R-free** | 0.2072 (0.3159) | 0.2206 (0.3461) | 0.2105 (0.2730) | 0.1929 (0.3072) |
| **CC(work)** | 0.959 (0.879) | 0.956 (0.819) | 0.952 (0.798) | 0.965 (0.811) |
| **CC(free)** | 0.949 (0.845) | 0.959 (0.750) | 0.925 (0.806) | 0.968 (0.784) |
| **Number of non-hydrogen atoms** | 6008 | 5656 | 11962 | 5681 |
| **macromolecules** | 5132 | 5054 | 10610 | 5070 |
| **ligands** | 31 | 30 | 142 | 109 |
| **solvent** | 845 | 572 | 1210 | 534 |
| **Protein residues** | 647 | 642 | 1347 | 641 |
| **RMS(bonds)** | 0.010 | 0.009 | 0.010 | 0.009 |
| **RMS(angles)** | 1.34 | 1.52 | 1.38 | 1.11 |
| **Ramachandran favored (%)** | 97.98 | 97.49 | 96.56 | 97.33 |
| **Ramachandran allowed (%)** | 1.71 | 2.19 | 2.91 | 2.35 |
| **Ramachandran outliers (%)** | 0.31 | 0.31 | 0.52 | 0.31 |
| **Rotamer outliers (%)** | 0.18 | 2.39 | 1.22 | 0.18 |
| **Clashscore** | 2.03 | 2.66 | 3.53 | 3.79 |
| **Average B-factor** | 17.43 | 35.01 | 28.11 | 19.83 |
| **macromolecules** | 15.66 | 34.08 | 27.08 | 18.61 |
| **ligands** | 26.46 | 60.20 | 54.31 | 33.34 |
| **solvent** | 27.89 | 41.96 | 34.11 | 29.45 |

Statistics for the highest-resolution shell are shown in parentheses.

^1^The final model contains residues 72-393 in chain A and residues 69-393 in chain B

^2^The final model contains residues 75-394 in chain A and residues 72-393 in in chain B

^3^The final model contains residues 49-396 in chain A, residues 70-393 in chain B, residues

64-394 in chain C and residues 53-396 in chain D. This structure is the most complete.

^4^The final model contains residues 75-395 in chain A and residues 75-394 in in chain B
