# Supplementary material for: Structure–function analysis of Lactiplantibacillus plantarum DltE reveals D-alanylated lipoteichoic acids as direct cues supporting Drosophila juvenile growth
Source: eLife. 2023 Apr 12;12:e84669. doi: 10.7554/eLife.84669 (PMC10241514; doi:10.7554/eLife.84669)
Supplement: Supplementary file 2. [file elife-84669-supp2.docx]

**Supplementary Table 2.** Proton and carbon chemical shifts of Glc, Gro and Ala constituents of LTA purified from *L. plantarum* WT.

**
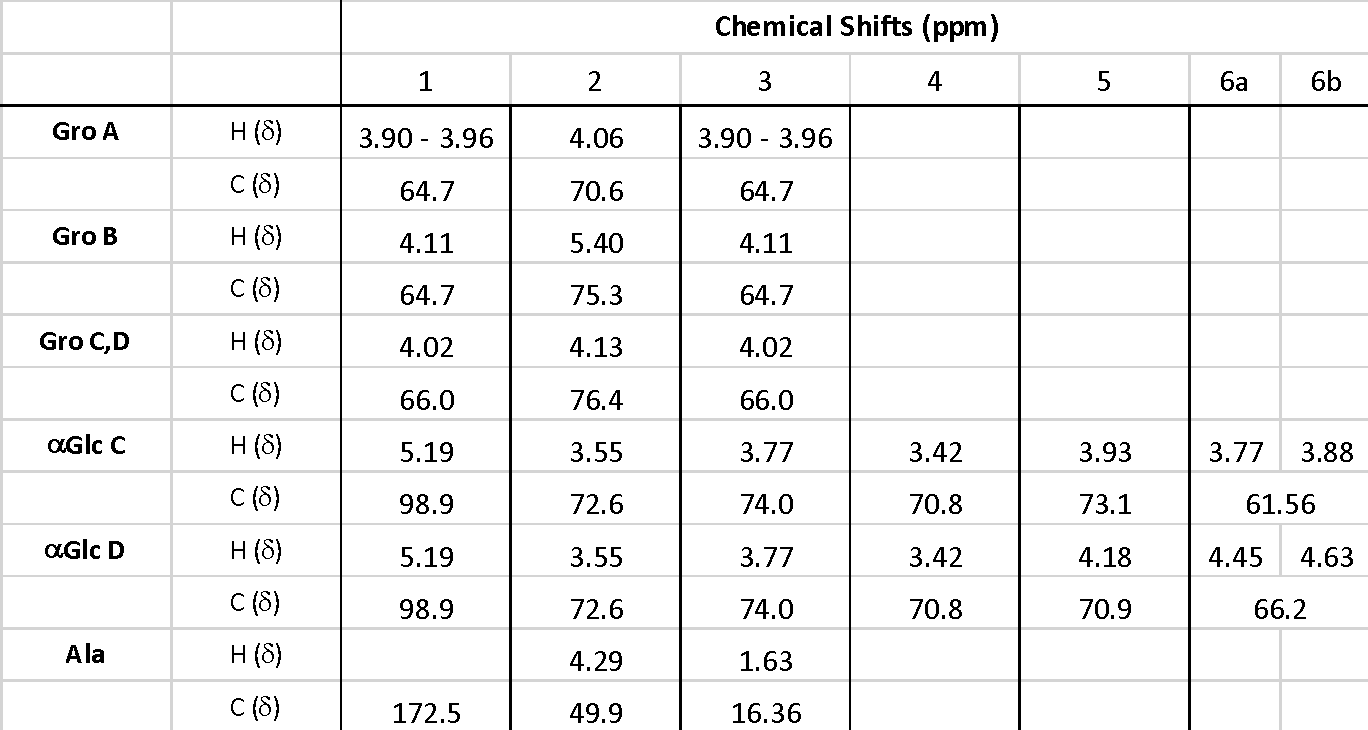
**
