# Supplementary material for: Structure–function analysis of Lactiplantibacillus plantarum DltE reveals D-alanylated lipoteichoic acids as direct cues supporting Drosophila juvenile growth
Source: eLife. 2023 Apr 12;12:e84669. doi: 10.7554/eLife.84669 (PMC10241514; doi:10.7554/eLife.84669)
Supplement: Supplementary file 3. [file elife-84669-supp3.docx]

**Supplementary Table 3.** Primers used in this study.

| Primer name | Sequence (5’→3’)^*^ | Reference |
| --- | --- | --- |
| XL01 | CTTGATATCGAATTCCTGCACTTGATTCAAAATCAAGAGACCCT | This study |
| XL02 | TACCATGCCTGATTAATCGAACTCGTATCAACTAAGGG | This study |
| XL03 | TCGATTAATCAGGCATGGTAATTTCTTCCTCCG | This study |
| XL04 | AGTGGATCCCCCGGGCTGCAACGTGCTCAGGCGTGTTGAA | This study |
| XL05 | CTTGATATCGAATTCCTGCAGACACCGGCATCCTTATTAA | This study |
| XL06 | CACAAATGATCCATTAAAAACCAAATAAATCATTGA | This study |
| XL07 | GGTTTTTAATGGATCATTTGTGCGTAACTCCCT | This study |
| XL08 | AGTGGATCCCCCGGGCTGCACCGAATCCACGTGCACTATA | This study |
| XL09 | AGTGGATCCCCCGGGCTGCAAACAGTACCAATCAGAAGAGGA | This study |
| XL10 | CTATAATTTAGTTTCTCATTTCTCAATTATCCCTTTCT | This study |
| XL11 | TTGAGAAATGAGAAACTAAATTATAGCAGTTAGTGT | This study |
| XL12 | CTTGATATCGAATTCCTGCAGTAACTGGTTTAAGATCAGCCGT | This study |
| XL13 | AGTGGATCCCCCGGGCTGCAGCCATGTTAATTGGTTTTCA | This study |
| XL14 | GAGCATCACTTTAACATAGTACCTTCCTTTAATTCGT | This study |
| XL15 | GTACTATGTTAAAGTGATGCTCGCTTAATAGATCG | This study |
| XL16 | CTTGATATCGAATTCCTGCATACGGTAGCGACCACGTCTC | This study |
| XL17 | TGGATCCCCCGGGCTGCAATGCGGCTTCAAAATCAAGGT | This study |
| XL18 | ATCGATAAAATTTGATACTTTGAATTGACTTTATTACGT | This study |
| XL19 | CAAAGTATCAAATTTTAGCAATTCAAAAGTCAATGACGG | This study |
| XL20 | TGATATCGAATTCCTGCACTGGTCAGGCAATCCGAAGT | This study |
| rp49f | GACGCTTCAAGGGACAGTATCTG | ^3^ |
| rp49r | AAACGCGGTTCTGCATGA | ^3^ |
| jon66ciif | AAACTGACCCCGGTCCAC | ^3^ |
| jon66ciir | CCTCCCAG CCGAT AGC | ^3^ |
| jon65Aif | CAACAACTACCAGGCTGGTG | ^3^ |
| jon65Air | GCCCTCATCGGAGGTCTT | ^3^ |

^*^Overlapping sequences for Gibson assembly are underlined.
