# Supplementary material for: Structure–function analysis of Lactiplantibacillus plantarum DltE reveals D-alanylated lipoteichoic acids as direct cues supporting Drosophila juvenile growth
Source: eLife. 2023 Apr 12;12:e84669. doi: 10.7554/eLife.84669 (PMC10241514; doi:10.7554/eLife.84669)
Supplement: Supplementary file 4. [file elife-84669-supp4.docx]

**Supplementary Table 4.** Bacterial strains and plasmids used in this study.

| **Strain or plasmid** | **Relevant characteristics** | **Reference or source** |
| --- | --- | --- |
| **Strain** |  |  |
| ***E. coli*** |  |  |
| TG1 | *supE hsd5h thi (∆lac-proAB) F’ (traD36 proAB-lacZ∆M15)* | ^4^ |
| GM1674 | *dam^-^dcm^-^ repA^+^* | ^5^ |
| ***L. plantarum*** |  |  |
| NC8 | Isolated from grass silage, plasmid free | ^6^ |
| *∆dltE* | NC8 strain deleted for *nc8_1738* (formerly *pbpX2*) | ^7^ |
| *∆dltXABCD* | NC8 strain deleted from *nc8_1737* to *nc8_1733* | ^7^ |
| *∆dlt_op_* | NC8 strain deleted from *nc8_1738* to *nc8_1733* | ^7^ |
| *∆ltaS* | NC8 strain deleted for *nc8_1125* | This study |
| *∆tagO* | NC8 strain deleted for *nc8_0646* | This study |
| *∆dacA1A2* | NC8 strain deleted for *nc8_2720* and *nc8_1122* | This study |
| *dltE^S128A^* | Knock-in of *dltE* mutated on S128 on *∆dltE* strain | This study |
| **Plasmids** |  |  |
| pG+host9 | Erm^r^, repATs | ^8^ |
