# Supplementary material for: Structure–function analysis of Lactiplantibacillus plantarum DltE reveals D-alanylated lipoteichoic acids as direct cues supporting Drosophila juvenile growth
Source: eLife. 2023 Apr 12;12:e84669. doi: 10.7554/eLife.84669 (PMC10241514; doi:10.7554/eLife.84669)
Supplement: Supplementary file 5. [file elife-84669-supp5.docx]

**Supplementary Table 5.** Primers used for *E. coli* plasmid constructions.

| **Number** | **Name** | **Sequence (5’ to 3’)** | **Reference** |
| --- | --- | --- | --- |
| 1 | 5-pPbpX2_extra_ | AGATATACCATGGCTACAGAGCGGCAAGCAGC | This study |
| 2 | 3-pPbpX2 _extra_ | TCGACTCGAGCTTAATATCACCCTTCATTAATTCCGTAAAAATCTTG | This study |
| 3 | 5-pPbpX2 _extra_ S128A | GTTGCTAGCTGGCATTATTCGC | This study |
| 4 | 3-pPbpX2 _extra_ S128A | GCGAATAATGCCAGCTAGCAAC | This study |
