# Supplementary material for: Structure–function analysis of Lactiplantibacillus plantarum DltE reveals D-alanylated lipoteichoic acids as direct cues supporting Drosophila juvenile growth
Source: eLife. 2023 Apr 12;12:e84669. doi: 10.7554/eLife.84669 (PMC10241514; doi:10.7554/eLife.84669)
Supplement: Supplementary file 6. [file elife-84669-supp6.docx]

**Supplementary Table 6.** Plasmids used in this study

| **Plasmid** | **Description and main characteristics** | **Source** | **Primers** |
| --- | --- | --- | --- |
| pET-28a(+) | T7 promoter, C-terminal 6×His, Kan^R^ | Novagen | - |
| pPbpX2 _extra_ | pET-28a(+) derivative encoding *L. plantarum* wild-type PbpX2 extracellular domain (residues 34-397) fused to a C-terminal (His)_6_ tag | This study | 1,2 |
| pPbpX2 _extra_ S128A | pET-28a(+) derivative encoding *L. plantarum* PbpX2 extracellular domain S128A mutant fused to a C-terminal (His)_6_ tag | This study | 3,4 |
